# Conformational Landscape, Polymorphism, and Solid Forms Design of Bicalutamide: A Review

**DOI:** 10.3390/molecules30183793

**Published:** 2025-09-18

**Authors:** Konstantin V. Belov, Ilya A. Khodov

**Affiliations:** G.A. Krestov Institute of Solution Chemistry of the Russian Academy of Sciences, Ivanovo 153045, Russia; kvb@isc-ras.ru

**Keywords:** Bicalutamide, non-steroidal antiandrogen, conformational polymorphism, supercritical fluids, solid dispersions

## Abstract

Bicalutamide (BCL), a clinically important non-steroidal antiandrogen, exhibits pronounced conformational polymorphism and complex solid-state behaviour that critically influence its physicochemical and biopharmaceutical properties. This review comprehensively integrates current computational and experimental findings on the structural features, polymorphic forms, and intermolecular stabilisation mechanisms of BCL. Key factors, including torsional flexibility, hydrogen-bond networks, π–π stacking, and fluorine–fluorine contacts, are examined with respect to polymorph stability, solubility, and dissolution kinetics. The review also synthesises recent advances in solid-state optimisation strategies—including co-crystals, solvates, amorphous forms, and solid dispersions—and explores the emerging role of supercritical fluid (SCF) technologies in particle engineering and dissolution enhancement. This work offers a framework for designing next-generation BCL solid forms with enhanced bioavailability and stability by connecting molecular insights with formulation approaches.

## 1. Introduction

Malignant neoplasms remain one of the most urgent global health challenges, ranking as the second leading cause of mortality worldwide after cardiovascular diseases [1,2,3]. Prostate cancer is among the most prevalent malignancies in men and the fifth most common cause of cancer-related death [4]. In its early stages, most prostate carcinomas are androgen-dependent [5]. The primary therapeutic strategy—superseding surgical castration [6]—involves the use of pharmacological agents (antiandrogens) to suppress the biological roles of testosterone and dihydrotestosterone (DHT) in tumour progression [7,8,9,10].

At the molecular level, the therapeutic performance of non-steroidal antiandrogens is governed not only by their affinity for the androgen receptor (AR) but also by their conformational flexibility and solid-state organisation. Structural analyses have shown that specific functional groups—such as the cyano moiety in BCL—stabilise receptor binding through hydrogen bonding and hydrophobic interactions, offering a blueprint for ligand optimisation [11]. Conformational polymorphism of BCL, particularly differences in the C(aryl)–C–S–C torsion angle between Forms I and II, influences lattice packing, thermodynamic stability, and dissolution behaviour [12,13].

Testosterone is converted into the more biologically active DHT by the enzyme 5α-reductase, which is predominantly localised in prostatic tissue [14]. Although both androgens stimulate the growth of normal and malignant prostate tissue, DHT is the principal driver of androgen-dependent tumour proliferation [15,16]. The first successful use of antiandrogens was reported by Drs. Charles Huggins and Clarence Hodges in 1941, for which Huggins was awarded the Nobel Prize [17,18]. Since then, medicinal chemistry efforts have refined pharmacophores through structure–activity relationship (SAR) studies, demonstrating that modifications to the aromatic anilide scaffold can fine-tune AR selectivity and shift the antagonist–agonist balance, with the incorporation of ferrocenyl groups emerging as a promising strategy [19]. On the other hand, the results of several studies [20,21,22] have shown that while prostate cancer progression is commonly associated with elevated DHT levels, there is evidence that some patients with low testosterone and low DHT may also develop the disease. Low androgen levels and an imbalance between testosterone, DHT, and oestradiol can have adverse effects in men and contribute to prostate pathology. Moreover, in the study by H. Klocker and colleagues [23], it was demonstrated that BCL, which acts as a “pure” antagonist, is nevertheless capable of exhibiting agonistic effects on AR transactivation activity in the later stages of therapy. In this context, research into antiandrogens represents a complex and multifaceted challenge. Antiandrogens are broadly divided into steroidal (e.g., cyproterone acetate, medroxyprogesterone acetate, and megestrol acetate) and non-steroidal classes (including nilutamide, BCL, and flutamide) [24]. Members of both groups, alone or in combination, are widely used in managing different stages of prostate cancer [25,26,27,28,29,30,31,32,33,34,35]. These compounds fall under Class II of the Biopharmaceutics Classification System (BCS) [36], characterised by low aqueous solubility and high membrane permeability, making them suitable candidates for solid-form optimisation [37,38,39]. Poor solubility, which contributes to variability in therapeutic exposure and adverse effects [40], underscores the need for analogues with improved dissolution profiles and reduced toxicity. In the case of BCL, the most common adverse effects are due to its pharmacological property of competitively blocking androgen receptors and include gynaecomastia, breast pain, fatigue, and reduced libido [41,42]. Over the past decade, intensified research has focused on modifying existing Class II drugs to enhance their biopharmaceutical performance [43,44]. In view of the above, it is precisely for BCL, as a pharmaceutically active ingredient and a promising antiandrogen, that the search for ways to develop new polymorphic (and other solid) forms is of particular interest.

Common strategies include micellar solubilisation [45,46], particle micronisation [47,48,49], complexation [50,51], supersaturating drug delivery systems (SDDS) [52,53], and the creation of solid forms such as polymorphs, co-crystals, crystal solvates, and salts [54,55,56,57]. These approaches aim to modulate intermolecular interactions—hydrogen bonding networks, π–π stacking, and halogen contacts—that define lattice stability and dissolution kinetics.

The search for and rational design of new solid forms, along with elucidation of nucleation mechanisms from solution, represents a promising route for imparting advantageous physicochemical and pharmaceutical properties. Factors limiting drug bioavailability include poor solubility in aqueous and buffered media [58,59,60,61], restricted membrane permeability [62,63], the need to stabilise specific solid forms [55,64,65,66], and tabletability constraints [67,68], among others [69,70]. The main barriers to progress are high time and cost demands; the development of a new drug or a modified form of an existing one can take up to 15 years [71,72,73]. Until recently, the discovery of new solid forms was largely empirical, relying on iterative trial-and-error combined with extensive physicochemical characterisation [74,75]. Current research is increasingly directed toward computational and experimental elucidation of molecular features that drive nucleation, enabling the prediction and targeted stabilisation of desired crystal forms.

This review synthesises and updates current knowledge on the molecular and supramolecular factors governing nucleation of BCL solid forms, identifying key structural motifs that stabilise its crystal lattice. Computational and experimental insights are integrated to guide structure-based optimisation of this clinically important non-steroidal antiandrogen.

## 2. Structural and Conformational Characteristics of Bicalutamide

### 2.1. Structural Features of Bicalutamide

The BCL molecule is a cornerstone of both classical and modern research. Understanding its chemical composition and spatial structure, based on the current body of scientific evidence, is not only essential but also a significant contribution to the field [76,77]. A single chiral centre—the asymmetric C10 atom—serves as one of the key structural motifs determining the biological activity of the molecule [78,79]. In the crystalline state, BCL exists as a racemic mixture of R and S isomers [80,81], whose physicochemical characteristics have been extensively characterised in several previous studies [82,83]. The separation of BCL isomers, particularly the R-isomer, which exhibits significantly higher binding affinity, is not merely a technical challenge but a critical task. This isomer accounts almost entirely for the antiandrogenic activity of the racemate [81]. The S-isomer, in contrast, is metabolised more rapidly, potentially resulting in an increased hepatic load. Similar trends have been reported for a series of BCL derivatives containing electrophilic groups synthesised under the direction of D.D. Miller [84]. The successful separation of these isomers in several analytical studies underscores the urgency and importance of this work [85,86,87]. However, most of the well-known solid forms, including those discussed in the present work, represent a racemic mixture containing both types of BCL isomers.

The work of A.T. Hagler et al. [88] highlights the particular importance of evaluating the spatial structure and conformational flexibility of BCL in relation to its macromolecular target—the AR. Upon binding testosterone and DHT, AR stimulates androgen production in tissues such as the prostate and muscle, promoting the proliferation of cancerous cells. C.E. Bohl et al. [89] resolved the AR W741L mutant ligand-binding domain (LBD) bound to R-BCL, showing a curved (“closed”) conformation, OH group donation, and intramolecular NH–SO_2_ hydrogen bonding. Structural features were linked to antagonistic activity. C. Bertucci et al. [90] delved into derivatives with higher AR affinity than R-BCL. They found a correlation with lipophilicity (LogP), where the compounds (R)-bic51 (substituted with a naphthyl fragment at atom C11) and (R)-bic59 (substituted with a (trifluoromethoxy)benzene fragment at atom C11) (LogP 4.765 and 4.552, respectively) exhibited stronger binding than R-BCL (2.154), whilst (S)-bic14 (bearing NH_2_ and CH_3_ groups at position C10) (2.197) displayed reduced affinity. Importantly, their study emphasised the role of SPR analysis in elucidating AR interactions, demonstrating that binding kinetics, rather than affinity alone, play a significant role in these processes.

Competitive inhibition of AR by antiandrogens such as BCL, as demonstrated by its significantly higher binding affinity and antiandrogenic activity, remains an effective, albeit temporary, therapeutic strategy against prostate cancer. Consequently, structural analysis of BCL in diverse environments is a high-priority objective in the development of next-generation AR antagonists.

### 2.2. Polymorphism, Conformational Classification, and Energetics

**Figure 1:**
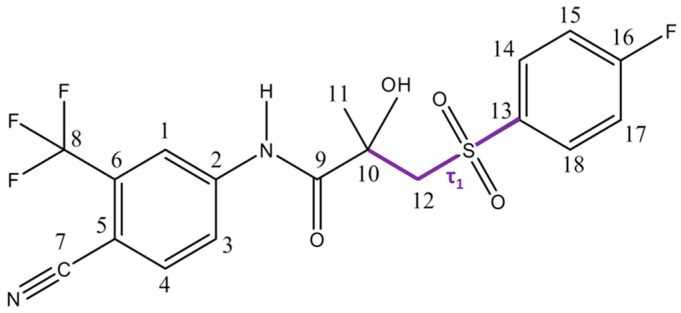
Structure of the BCL molecule with atom numbering used for discussion of bond angles, bond lengths, and internuclear distances. The violet lines mark the τ_1_ angle.

A comprehensive conformational search by I.A. Khodov [77] was conducted with meticulous attention to detail, revealing that known polymorphic forms of BCL differ primarily in the torsional angle τ_1_ (C10–C12–S–C13), with values of −88.3° and 72.5° for Forms I and II, respectively [12]. The study focused on variations directly associated with this torsional angle (Figure 1).

**Figure 2:**
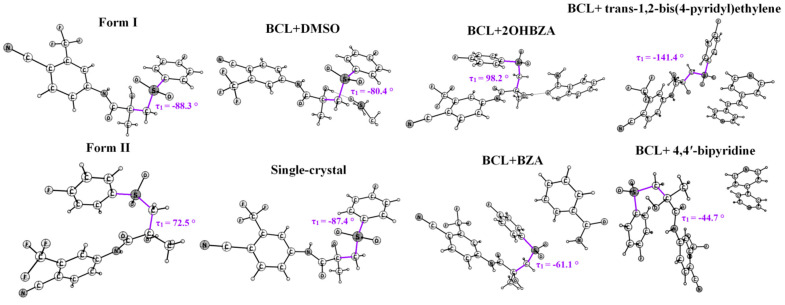
BCL molecular structures in various solid forms, with τ_1_ values (violet) indicated. The abbreviations BZA and 2OHBZA stand for benzamide and salicylamide.

Beyond the two polymorphs, solid-state forms of BCL include a solvate with dimethyl sulfoxide (DMSO) [91], four co-crystals [92,93], a bioactive form bound to AR [89], and a single-crystal structure [94] (Figure 2). The torsional angle τ_2_ (N(H)–C9–C10–C12) remains essentially constant across all known solid forms, making it another critical parameter in pharmaceutical design [95]. These findings have direct implications for the practical design of pharmaceuticals.

**Figure 3:**
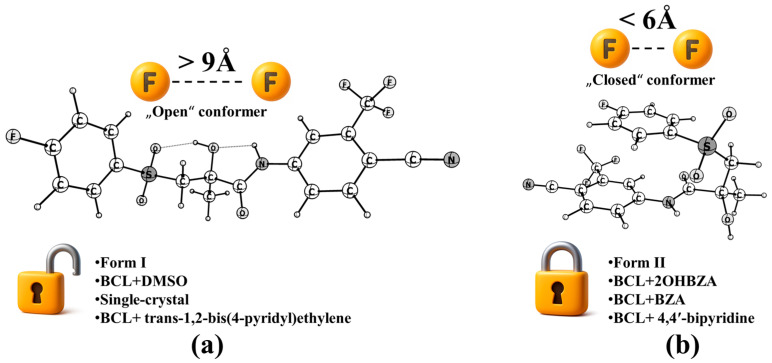
Examples of “open” (**a**) and “closed” (**b**) conformers, alongside solid forms composed of each type.

Conformational classification distinguishes “closed” and “open” folds of the molecule, which are defined by the relative orientation of the substituted aromatic rings. In the “closed” form, peripheral fluorine atoms are co-directional with interatomic distances < 6 Å; in the “open” form, they are anti-parallel with distances > 9 Å [96] (Figure 3). This distinction is important as it provides insights into the structural flexibility and potential reactivity of BCL. An alternative naming scheme, “syn-” and “anti-”, based on τ_1_ values, was proposed by P.V. Bharatam [80], but it is insufficient to capture the diversity of possible conformers. D.R. Vega et al. [12] first identified τ_1_ as the principal structural descriptor separating “open” and “closed” folding.

Energy profiling by Khodov et al. revealed ten conformers with relative electronic energies ranging from 0 to 31.02 kJ/mol. The lowest-energy conformers, BCL-1 and BCL-2, both belong to the “closed” type. This is consistent with Boltzmann-weighted free energy analyses in both the gas phase and in solvent, where “closed” conformers predominate.

On the other hand, M. Pu et al. [97] identified only two low-energy conformers (A and B) using B3LYP/6-311+G** and the polarisable continuum model (PCM) to account for solvent effects. These conformers, A and B, seem to correspond to different types and, according to the authors, are in good agreement with the molecular structures forming distinct polymorphs. However, upon closer inspection of M. Pu’s results, this assertion does not seem reliable, and comparison of geometric characteristics often does not allow for an unambiguous correspondence between the obtained conformers and the BCL structures forming polymorphic Forms I and II. The discrepancies in these studies are not only intriguing but also significant, as they could potentially lead to a better understanding of BCL’s behaviour.

At the same time, their calculated interconversion barrier (5.78 kJ/mol) is inconsistent with Khodov’s results (>25.59 kJ/mol for the “closed” to “open” transition), suggesting a possible underestimation of torsional rigidity in earlier models. Such an underestimation could lead to inaccurate predictions of BCL’s behaviour in different environments, underscoring the importance of our current research. The need for accurate modelling in our research is not just a requirement but a necessity, as it could significantly impact our understanding of BCL’s behaviour.

### 2.3. Solvent Effects, Non-Covalent Interactions, and Stabilisation Mechanisms

To assess medium effects, Khodov et al. applied the integral equation formalism polarisable continuum model (IEFPCM) [98,99]. Gibbs solvation energies in CHCl_3_ and DMSO were −41.26 ± 6.03 and −58.44 ± 8.99 kJ/mol, respectively, with minimal variation across conformers, suggesting that solvent polarity has limited influence on conformer ranking. G.L. Perlovich et al. [100] provide further thermodynamic analysis. Historically, hydrogen bonding was considered the primary stabilisation motif [12]. More recently, the quantum theory of atoms in molecules (QTAIM) analyses [101,102] have revealed a more nuanced picture: “closed” conformers gain additional stability from multiple weak interactions between aromatic fragments. In contrast, “open” conformers rely mainly on hydrogen bonds in the aliphatic linker region. Among three stable dimers identified in [80], D2 (“closed”) had the most negative interaction energy (−134.47 kJ/mol) compared to “open”-form dimers D1 (−102.02 kJ/mol) and D3 (−109.08 kJ/mol). Interestingly, hydrogen bonds contributed less to D2 stability (−8.2 kJ/mol) than to D1/D3 (−35.3 and −39.4 kJ/mol), suggesting that weaker, cumulative non-covalent forces dominate in “closed”-form dimers.

**Figure 4:**
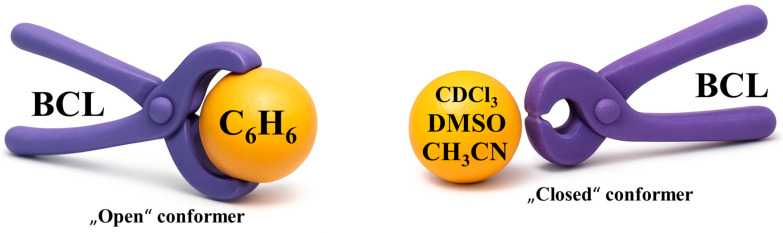
Localisation of different solvent molecules relative to BCL.

Solvent-inclusion calculations [103] revealed that only benzene molecules efficiently localise between the substituted aromatic rings of BCL, partially opening the fold (maximum deformation energy: 35.47 kJ/mol). Chloroform, DMSO, and acetonitrile induced minor structural changes without whole opening, instead causing planar displacements of the rings (Figure 4).

QTAIM confirmed strong intermolecular BCL–benzene contacts in “open” structures, while other solvents preserved “closed”-form non-covalent patterns. These findings underscore the importance of cumulative weak interactions—not just hydrogen bonding—in stabilising small-molecule conformations in both the solid state and solution.

## 3. Experimental Methods in the Structural and Physicochemical Characterisation of BCL

### 3.1. Analytical Techniques Applied to BCL

It is essential to assess the relationship between insights into BCL polymorphs and weak intermolecular forces and experimental data. For example, the quantum-chemical prediction of enhanced stability for the “closed” conformation through intramolecular NH–SO_2_ hydrogen bonding, as well as the calculated propensity for π–π stacking in dimeric motifs, suggests structural features that solid-state analytical techniques can directly probe. Similarly, the simulated reduction in crystallisation tendency for specific conformations provides a framework for interpreting the phase transformation behaviour observed in practice.

A broad range of experimental methods has been used to confirm and extend computational findings on BCL, including nuclear magnetic resonance (NMR) spectroscopy [76,77,96,103,104], infrared (IR) spectroscopy [76,105,106], Raman spectroscopy [12,107,108], vibrational circular dichroism (VCD) [76], scanning electron microscopy (SEM) [109,110], X-ray powder diffraction (XRPD) [12,93,111], differential scanning calorimetry (DSC) [93,100,110,112], broadband dielectric spectroscopy (BDS) [105,112], fluorescence spectroscopy [110], and surface plasmon resonance (SPR) [90].

### 3.2. Polymorphism and Crystalline Structures

The groundbreaking research by D.R. Vega et al. [12] unveiled the two primary crystalline states of BCL—polymorphs I and II. Their discovery of the high conformational mobility leading to conformation-dependent polymorphism [113,114,115,116,117], reduced crystallisation propensity [118], and facile amorphisation is a significant contribution to the field.

The patent literature [119,120] describes two crystalline and one amorphous form. Form I crystallises from ethanol at room temperature, a condition that is more easily achievable in practical settings. In contrast, Form II requires seeded crystallisation at 40 °C, a condition that may be more challenging to replicate. The amorphous form is produced by quenching molten Form I.

The thermodynamic properties of the polymorphs and their dissolution kinetics should be considered in the context of accurate solubility determinations. The first qualitative investigation of the solubility of the two polymorphic forms of BCL was reported in [12]. The authors’ inference that Form II is approximately 2.5 times more soluble than Form I, based on the relative intensities of the diagnostic peaks of the two forms in ultraviolet–visible (UV–vis) spectra, is a significant discovery in the field.

A more systematic and quantitative study of the solubility and thermodynamic parameters of a broader set of BCL solid forms was subsequently presented in [91]. In this work, saturated aqueous solutions of BCL were prepared and maintained at 298.15 K (isothermal saturation method) until equilibrium was achieved. The residual solid phase was then removed by isothermal filtration and centrifugation, and the equilibrium solubility was quantified spectrophotometrically [121]. The solubility values (m.f.) obtained were 1.38 × 10^−7^ for Form I and 3.38 × 10^−7^ for Form II, the latter reverting to Form I within 110 h.

The same study further assessed the thermodynamic parameters and dissolution kinetics of the BCL solid forms, including the two polymorphs. Dissolution kinetics were evaluated in aqueous medium, with solubility measurements taken at defined time intervals. The results demonstrated that Form II progressively transforms into the more stable Form I, while the amorphous phase also tends towards the stable crystalline state. Thermodynamic functions of the phase transitions were calculated, including enthalpy (ΔH) and entropy (ΔS) changes for the transformations between polymorphic and amorphous states (see Section 3.4).

It is noteworthy that the solubility data for the polymorphic forms are in close agreement with the earlier qualitative findings on the relative solubility of Forms I and II.

Each polymorph contains one molecule in the asymmetric unit (Z′ = 1). In Form I, the τ1 (C10–C12–S–C13) torsion angle is −88.3°, compared with −72.5° in Form II. Both forms are stabilised by weak hydrogen bonds, with donor sites on the NH and OH groups, but differ in C=O acceptor interactions: H3 in Form I versus H1 in Form II, reflecting C3–C2–N(H)–C9 torsion angles of −28.5° and −164.4°, respectively (see Table 1).

Form I molecules in “open” conformation form extended O–H…O(S) hydrogen-bonded chains; “closed” conformations in Form II favour dimer formation via π–π stacking. Raman spectra differentiate the forms: Form I shows a single maximum at 150 cm^−1^; Form II exhibits bands at 600, 1450, and 1700 cm^−1.^

### 3.3. Amorphous State and Phase Transformation Behaviour

Under polarising microscopy equipped with a heating stage, the amorphous phase of BCL undergoes a thermally induced transition to Form II at approximately 105 °C. Remarkably, even in the absence of external thermal input, this metastable Form II spontaneously forms from the amorphous phase at ambient conditions within about one week. This observation highlights the amorphous form’s liability and the system’s kinetic instability. Mechanical activation, like grinding or milling, accelerates polymorphic transformations. Z. Németh et al. [122] showed that gentle mechanical treatment can create seeds of Form I in the amorphous matrix, facilitating the transformation to the more stable polymorph at elevated temperatures [122]. This behaviour is not unique to BCL; similar mechanical activation effects have been observed in other compounds such as ursodeoxycholic acid, where mechanical stress induces surface crystallisation or creates supersaturated intermediates that promote phase change [123]. Such susceptibility to mechanically induced transformations necessitates stringent control over environmental conditions and handling protocols in both research and industrial manufacturing settings, particularly when working with metastable or amorphous pharmaceutical solids.

### 3.4. Solvates and Co-Crystals

G.L. Perlovich et al. [91] unveiled a significant milestone in our understanding of BCL crystal forms. They reported the first BCL+DMSO crystal solvate (1:1, FAHFIG) and meticulously measured enthalpies of solution (Δ_sol_H_298_) for Form I (9.6 ± 0.3 kJ/mol), Form II (4.1 ± 0.2 kJ/mol), the amorphous state (–14.5 ± 0.3 kJ/mol), and the solvate (22.0 ± 0.2 kJ/mol). The derived transition enthalpies were equally enlightening: I → II (5.5 ± 0.5 kJ/mol), I → amorphous (24.1 ± 0.6 kJ/mol), and II → amorphous (18.6 ± 0.5 kJ/mol). The solubility values for the amorphous state and the solvate were measured in accordance with the methodology described in Section 3.2. The values amounted to 3.63 × 10^−7^ for amorphous BCL; the solvate peaked at approximately 4.5 × 10^−7^ after 4.5 h, then declined as Form I emerged.

M. J. Zaworotko et al. [92] introduced a remarkable set of 17 co-crystals, each with distinct properties, two of which correspond to BCL co-crystals with 4,4′-bipyridine (m.p. 163 °C) and trans-1,2-bis(4-pyridyl)ethylene (m.p. 159 °C) (see Table 2).

G.A. Perlovich et al. [93] further expanded our knowledge with their description of BCL–benzamide and BCL–salicylamide (1:1), both featuring tetrameric H-bonded motifs with “closed” BCL conformations. The melting points of these co-crystals were 132.4 °C and 157.1 °C, respectively, versus 193.0 °C for pure BCL. In a pH 7.4 buffer, both displayed a rapid 5–7-fold increase in solubility within 20 min, followed by precipitation (“spring–parachute” effect).

### 3.5. Solid Dispersions and Carrier-Based Systems

Given its very low aqueous solubility [91,100], BCL has been formulated into solid dispersions (SDs) with polymeric or protein carriers. F. Ren et al. [106] demonstrated the potential of BCL–PVP K30 SDs (1:3, 1:4, 1:5) in improving drug dissolution. Their solvent evaporation method resulted in amorphisation at higher polymer content, confirmed by DSC, PXRD, and FT-IR. The dissolution rate of 1:5 SDs reached an impressive ~ 98% in just 10 min. J. Szczurek et al. [112] studied 1:2 and 2:1 BCL–PVP SDs via BDS and DSC, finding stable amorphous phases and identifying C=O (PVP) interactions with BCL donor–acceptor centres. F. Tres et al. [108] delved into the transformation process of BCL–copovidone VA64 SDs. Their examination, which involved hot-melt extrusion, real-time Raman mapping, and rotating disk dissolution, revealed a fascinating process. The 5% SD dissolved in 85 min. In comparison, 50% SDs remained stable >3000 min, forming a hydrophobic BCL shell that transformed from amorphous to Form II, then to Form I. J. Szafraniec-Szczęsny et al. [111] assessed low polymer concentrations in amorphous BCL stabilisation (ball milling and spray drying), finding morphological changes from hexagonal crystals to irregular aggregates and nanospheres. DSC revealed a single Tg in all cases, consistent with molecular dispersions. S. Mandal et al. [109] prepared BCL–PLGA nanoparticles by solvent evaporation. FT-IR, XRPD, and DSC confirmed the absence of chemical interaction; release was biphasic (58% in 24 h, 98% in 120 h) and fit the Higuchi diffusion model (R^2^ = 0.9991) F. Ren et al. [110] used bovine serum albumin (BSA) to reduce BCL particle size to 1–10 μm via steric hindrance and hydrophobic/hydrogen-bond interactions (–OH, –NH, –CN, –SO_2_). XRPD and DSC indicated Form I → Form II recrystallisation; dissolution exceeded 93% in 20 min versus 20% in 60 min for unmodified BCL.

### 3.6. Studies on BCL Conformation in Solution

Characterising BCL’s behaviour, particularly its molecular conformation in SDs, is challenging due to conformation-dependent polymorphism, which allows for comparisons between solution-phase and crystalline-state packing. This structural behaviour, a significant focus of extensive early studies, is crucial for designing new and optimising existing pharmaceutical solids [124,125,126,127,128,129,130,131]. These early studies have provided a detailed review of polymorphism in small-molecule drugs and its role in nucleation, as presented by Gong et al. [117].

Despite extensive data on solid-state forms, the studies of BCL molecular structure in solution remain limited, highlighting the need for further research. Early work [76] investigated two BCL derivatives—N-[4-nitro-3-(trifluoromethyl)phenyl] -3-(4-fluorophenyl)sulphinyl-2-hydroxy-2-methylpropanamide and its 4-cyano analogue—using NMR, IR, and VCD spectroscopy. ^13^C NMR chemical shifts (B3LYP/6-31 G (d, p)) aided in identifying dominant diastereomers despite signal overlap. IR and VCD spectra provided characteristic fingerprints of different conformers, revealing solvent effects: polar solvents favoured “extended” conformations, whereas non-polar solvents stabilised “U-shaped” ones.

A later comprehensive study by Rams-Baron et al. [105] examined tautomerism in amorphous BCL using IR, BDS, and NMR. This comprehensive study delved deep into the tautomerism in amorphous BCL, providing a thorough understanding of the subject. Density functional theory (DFT) calculations identified two proton-transfer mechanisms between the amide nitrogen (N1) and carbonyl oxygen: intramolecular (mechanism I) and intermolecular (mechanism II), with forward/reverse activation energies of 160/116 and 181/51 kJ/mol, respectively. IR spectra indicated the presence of the less stable imidic acid tautomer in quenched amorphous samples. BDS data above T_g_ (328 K) showed a shift towards the more stable amide form, with half-lives of 18 min at 335 K and ~70 min at 298 K. Activation energies from BDS (106 kJ/mol) matched DFT predictions for mechanism I. NMR lacked resolution to distinguish tautomers, but the amide form was found to predominate at room temperature.

### 3.7. Quantitative Analysis of Conformer Populations: Concentration Dependence and Influence of Solvent Acceptor Number

Khodov et al. [77,96,103,104] quantified the proportions of two conformational families—“open” and “closed”—in solution. Using ^1^H, ^13^C, and 2D NMR (^1^H–^13^C HSQC/HMBC, ^1^H–^1^H TOCSY/NOESY) in CDCl^3^ and DMSO-d^6^, the diagnostic H12b–H14/18 interproton distances were 4.21 Å (“closed”) and 3.35 Å (“open”). NOESY with the isolated spin-pair approximation (ISPA) model yielded “open”/“closed” ratios of 22.7/77.3% in CDCl_3_ and 59.8/40.2% in DMSO-d_6_, consistent with earlier observations [76] that polar solvents stabilise the “open” form. One-dimensional NOESY gave comparable values (61/39% in DMSO-d^6^). The BCL+DMSO solvate [91] showed a 3.16 Å H12b–H14/18 distance, corresponding to an “open” conformation.

These experimental results, which differ from the free-energy predictions in Section 2 that universally favoured the “closed” form, underscore the urgent need for direct experimental validation. This highlights the critical importance of further investigation into drug polymorphism and molecular conformation.

**Figure 5:**
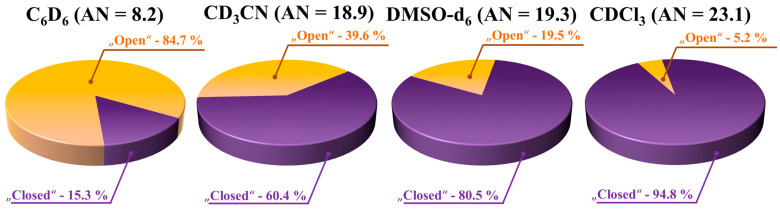
Circular diagrams illustrating the distribution of the proportions of the “open” and “closed” conformers of BCL in four solvents, derived from the analysis of experimental NOESY data.

In DMSO-d_6_, higher BCL concentrations shifted the equilibrium towards the “closed” form; ratios changed from 30/70% at 0.86 M to 63/37% at 2.16 M [104]. At high concentrations, the system may represent a pre-nucleation state, with the “closed” form linked to metastable Form II. Further analysis [103] examined saturated BCL in deuterated benzene (C_6_D_6_), trideuteroacetonitrile (CD_3_CN), deuterated dimethyl sulfoxide (DMSO-d_6_), and deuterated chloroform (CDCl_3_). H12b–H14/18 distances and conformer ratios were as follows: C_6_D_6_ (15.3% “closed”, AN = 8.2), CD_3_CN (60.4%, AN = 18.9), DMSO-d_6_ (80.5%, AN = 19.3), and CDCl_3_ (94.8%, AN = 23.1) (see Figure 5). These data contradicted the assumption that polarity alone controls conformer ratios; instead, Gutmann’s acceptor number (AN) [132,133] correlated directly with the proportion of “closed” conformers.

### 3.8. Summary and Implications for Further Research

Integrating computational and experimental findings, the conformer population distribution of BCL is determined not only by solvent polarity and concentration but also by specific weak interactions such as intramolecular and intermolecular hydrogen bonding, fluorine–fluorine contacts, and C–H···π interactions. These interactions may not only stabilise certain conformers but also dictate the arrangement of molecules in the solid state, known as “packing motifs”, influencing the resulting crystal form and its properties. However, for a more detailed understanding of the kinetic mechanisms of BCL nucleation, the results of additional experimental studies over a wide range of concentrations would be helpful, for instance, in low-polarity solvents such as chloroform or benzene.

To date, no detailed experimental studies have addressed the conformational behaviour of BCL in SCF media—an important gap in the literature. This is especially significant considering the potential of SCFs, such as supercritical CO_2_, for solvent-free processing, polymorph control, and particle engineering. Given the proven influence of solvent and interaction types on conformer populations and polymorph selection, it is crucial that future studies explore how the tuneable properties of SCFs—such as density, polarity, and hydrogen bonding capability—can be leveraged to favour specific BCL conformers or polymorphs selectively. The following section addresses this underexplored yet promising area, outlining key experimental strategies and findings that could inform BCL behaviour in supercritical environments.

## 4. Bicalutamide in Supercritical Fluid Media

The SCF state, first observed by Charles Cagniard de la Tour in 1822 [134], underpins a range of modern, environmentally friendly, and efficient processing technologies [135,136,137,138,139,140,141,142]. SCFs, with their unique combination of gas-like viscosity, liquid-like density, and solvating power [143,144,145], offer a fascinating realm of possibilities. They boast low toxicity and rapid removal via depressurisation [146,147], and their properties can be finely tuned through control of pressure and temperature [148]. Among their diverse applications, SCFs are widely used for micronisation, a process that can markedly improve the dissolution rate and hence the bioavailability of poorly soluble active pharmaceutical ingredients (APIs) [149,150,151,152]. The potential of SCF technologies to significantly enhance dissolution rates is an exciting prospect in pharmaceutical processing. Micronisation approaches are typically classified according to the solubility of the target in the SCF:

- APIs readily soluble in the SCF are processed by methods such as rapid expansion of supercritical solutions (RESS) [153,154,155,156].
- Poorly soluble compounds are treated via supercritical anti-solvent (SAS) precipitation [157,158,159].

One of the most crucial steps in the process selection is determining a compound’s solubility in the chosen SCF. This forms the cornerstone of the entire process selection, underscoring its importance and the need for careful consideration.

### 4.1. Initial Solubility Studies

The groundbreaking solubility data for BCL in SCF-like conditions, a first of its kind, were reported by Foster et al. [160]. They measured the solubility of the API in subcritical water (SBCW) at a pressure of 5.5 MPa across a temperature range of 110–170 °C. According to their results, the solubility of BCL increased exponentially with temperature—from 0.79 × 10^−4^ M at 110 °C to 6.24 × 10^−4^ M at 170 °C—demonstrating an approximately eightfold increase. This pronounced enhancement confirms the strong temperature dependence of SBCW solubilisation mechanisms. It not only suggests the feasibility of using SCF-based approaches to overcome BCL’s poor aqueous solubility, a key limitation to its oral bioavailability, but also opens up new possibilities for drug development.

While these SCF-based formulations do not replace direct solubility measurements in SCF solvents like CO_2_, they reinforce the thermodynamic and kinetic benefits of SCF processing in enhancing the solubilisation potential of BCL. Overall, they highlight the importance of SCF and SBCW systems in enhancing BCL bioavailability through solubility modulation.

### 4.2. scCO_2_ Processing of BCL Solid Dispersions

**Figure 6:**
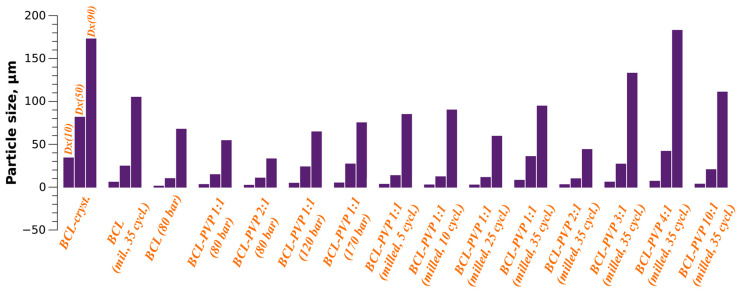
Particle size distribution according to Jachowicz et al. [154] for BCL forms obtained by milling and SCF processing.

Among SCFs, supercritical CO_2_ (scCO_2_) stands out as the most widely used due to its mild critical parameters (31.1 °C, 7.38 MPa). Its potential in pharmaceutical sciences is vast and promising, sparking intrigue and inspiration among researchers. Work by Jachowicz et al. [161] compared BCL–PVP solid dispersions prepared by ball milling and by scCO_2_ processing. Ball milling fully amorphised BCL, whereas scCO_2_ reduced crystallinity without complete amorphisation. Particle size decreased from 150 μm (raw) to <100 μm (processed) in both methods. Dissolution testing revealed that milling increased aqueous apparent solubility from 3.7 mg/mL to 79.2 mg/mL (21-fold), whereas scCO_2_ processing achieved solubilities of 12.1–19.1 mg/mL. At the same time, it was established that no solubility enhancement effect was observed when analysing the system based on BCL physically mixed with PVP. All particle size distributions of BCL obtained from the analysis in the study [161] are presented as a diagram in Figure 6.

Thus, milling gains derive mainly from amorphisation, whereas SCF gains reflect the process of reducing the particle size to a few micrometres, known as “micronisation”. However, mechanically amorphised BCL proved unstable, requiring stabilisation strategies, such as increasing carrier fraction [162]. A subsequent study [163] examined the impact of tableting on BCL–PVP SDs. Dissolution from tablets reached ~70% for milled SDs, ~36% for scCO_2_-processed SDs, and < 20% for physical mixtures and raw BCL. Compression partially amorphised even crystalline materials; however, the presence of PVP K-29/32 in milled SDs effectively prevented recrystallisation during storage, providing reassuring stability to the process.

### 4.3. Alternative Polymer Matrices

The same group also investigated SDs produced using SCF technology with two hydrophilic carriers: polyethylene glycol 6000 (PEG 6000) and Poloxamer® 407 (PLX 407) [164]. The resulting SDs demonstrated distinct particle size distributions; PLX 407-based SDs formed finer particles (≤100 μm), whereas PEG 6000-based systems showed a broader range (50–250 μm). Despite this difference, both systems achieved nearly identical and significantly improved dissolution rates: 74.80 ± 1.66% for PEG 6000 and 77.43 ± 6.01% for PLX 407 within one hour, representing an approximately ninefold enhancement over the crystalline form of BCL, which reached only 8.85 ± 1.02%.

### 4.4. Conformational Analysis in scCO_2_

Until recently, no molecular-level structural analysis of BCL in SCFs had been reported. In 2025, Khodov et al. [165,166] used nuclear Overhauser effect (NOE) spectroscopy to quantify “open” and “closed” conformers in scCO_2_. At 45 °C/9 MPa, the ratio was 80.9%/19.1%; at 55 °C/12.5 MPa, it shifted to 62.7%/37.3%. This suggests that increasing temperature disrupts intra- and intermolecular interactions, stabilising the “closed” form. The authors emphasised the need for further combined computational and experimental studies to elucidate SCF effects on BCL conformation, highlighting the potential for future research in this area.

### 4.5. Integration of SCF Processing Insights into Advanced Formulation Strategies

Section 4 shows that SCF technologies effectively produce solid-state forms of BCL with tailored particle sizes and morphologies. To translate these laboratory results into effective pharmaceuticals, a multifaceted approach is needed: (i) correlating SCF parameters with BCL’s outcomes, (ii) using kinetic and thermodynamic modelling for stability predictions, and (iii) integrating SCF methods into hybrid formulations—like combining SCF micronisation with polymeric dispersions—to enhance dissolution while ensuring stability. Establishing these connections can elevate SCF processing from an exploratory method to a reliable, scalable, and regulatory-compliant tool in BCL formulation science. This is especially relevant as SCF technologies can induce amorphous forms or metastable polymorphs, which may revert over time without proper stabilisation. Emerging work in particle design confirms that SCF can reproducibly control these parameters when integrated with real-time analytical techniques such as Raman spectroscopy or X-ray diffraction and formulation science methods like hot-melt extrusion or spray drying [167,168].

The next phase of development should explore embedding SCF into multi-component formulation systems. For example, co-crystallisation and polymer-based dispersions that incorporate SCF-processed BCL could combine rapid dissolution with mechanical and thermal robustness. These hybrid approaches align well with pharmaceutical regulatory requirements, which increasingly emphasise reproducibility and scalability of particle engineering technologies [169], providing reassurance about their compliance. By systematically building these correlations and integrating SCF technologies into formulation pipelines, what is now an exploratory methodology could become a central, scalable, and regulatory-compliant tool in BCL product development and beyond, sparking excitement about the future possibilities.

## 5. Conclusions

The present work provides a comprehensive review of the current state of research concerning the design of solid forms of bicalutamide, as well as their thermodynamic characteristics. The review, which includes an introduction and three chapters, meticulously examines experimental and computational data. It particularly emphasises the interplay between conformational flexibility, polymorphic variability, and the stabilising influence of diverse non-covalent interactions within the solid state, which collectively determine the structural and physicochemical behaviour of BCL. Conformational polymorphism, mainly determined by the C10–C12–S–C13 torsion angle, results in different “open” and “closed” conformers, each of which is associated with characteristic physicochemical properties. High-level quantum-chemical and QTAIM analyses demonstrate that, contrary to earlier models emphasising hydrogen bonding as the principal stabilising factor, the cumulative effect of weaker interactions—including π–π stacking, C–H···π contacts, and fluorine–fluorine interactions—can contribute more significantly to lattice energy, particularly in “closed” conformers.

In the second chapter, particular attention is given to identifying the stabilisation patterns of various BCL molecular structures that are part of solid forms. Experimental NMR investigations provide evidence that solvent environment and solute concentration substantially influence conformer population distributions, with Gutmann’s acceptor number emerging as a superior predictive descriptor compared to solvent polarity. This observation suggests that solvation dynamics and pre-nucleation equilibria should be incorporated into polymorph screening and crystallisation process design. The third chapter of this review provides comprehensive information on the established approaches to solid-state modification, including amorphisation, co-crystallisation, and polymer-assisted solid dispersions, which have demonstrated significant improvements in BCL dissolution rates, in some cases exceeding a tenfold increase. Furthermore, the fourth chapter compiles research data on solid forms of BCL in SCF environments. SCF processing is a useful method for adjusting particle size and surface properties while maintaining desired shapes and minimising unnecessary phase changes.

Nevertheless, conformational rearrangements and subtle lattice reorganisations detected under SCF conditions demonstrate that these processes can also induce shifts in the solid-state energy landscape. Such transformations may influence dissolution kinetics, mechanical properties, and ultimately bioavailability. Therefore, the integration of high-level computational modelling capable of simulating molecular conformations, lattice energies, and nucleation pathways together with in situ experimental monitoring under SCF conditions is essential for predicting and controlling structural evolution across a range of thermodynamic regimes. This combined approach will be critical for translating SCF-based methods into robust, reproducible, and industrially scalable pharmaceutical manufacturing processes for BCL.

Progress in BCL optimisation will rely on coupling predictive nucleation modelling, solvent–solute interaction mapping, and hybrid formulation platforms—particularly SCF-assisted co-crystallisation and polymeric stabilisation—with regulatory-compliant scale-up and stability-assured manufacturing. Such integrative methodologies will be essential for delivering BCL solid forms with optimised bioavailability, structural stability, and manufacturability.

## Figures and Tables

**Table 1:** Geometrical characteristics of molecular structures: values of dihedral angles, types of hydrogen bonds, solubility characteristics, and images of molecular structures forming polymorphic forms (I and II) of BCL.

	Conf.	Z’	τ_1_ (C10–C12–S–C13)	X–H···Y,	τ_1_ (C3–C2–N(H)–C9)	χ, M	Structure
Form I (hydrogen-bonded chains)	“Open”	1	–88.3°	N–H···O(H) O–H···O(S) C–H3···O=C	−28.5°	1.38 × 10^−7^	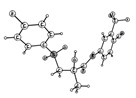
Form II (π–π stacking)	“Closed”	1	–72.5°	N–H···O(H) O–H···O(S) C–H1···O=C	−164.4°	3.38 × 10^−7^	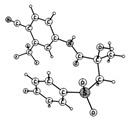

**Table 2:** Thermodynamic characteristics of polymorphic and solvate forms of BCL based on the literature data.

	Δ_sol_H_298_, kJ/mol	ΔH_tr_, kJ/mol	m.p., °C
Form I	9.6 ± 0.3	I → II 5.5 ± 0.5	193
Form II	4.1 ± 0.2		
Amorphous	–14.5 ± 0.3	I → Am. 24.1 ± 0.6 II → Am. 18.6 ± 0.5	-
BCL+DMSO	22.0 ± 0.2	-	115
BCL+4,4′-bipyridine	-		163
BCL+trans-1,2-bis(4-pyridyl)ethylene			159
BCL+benzamide			132
BCL+salicylamide			157

## Data Availability

Not applicable.

## Abbreviations

The following abbreviations are used in this manuscript:

BCL	Bicalutamide
SCF	Supercritical fluid
DHT	Dihydrotestosterone
AR	Androgen receptor
SAR	Structure–activity relationship
BCS	Biopharmaceutics Classification System
SDDS	Supersaturating drug delivery systems
LBD	Ligand-binding domain
LogP	Logarithm of the octanol–water partition coefficient
PCM	Polarisable continuum model
IEFPCM	Integral equation formalism polarisable continuum model
DMSO	Dimethyl sulfoxide
QTAIM	Quantum theory of atoms in molecules
NMR	Nuclear magnetic resonance
IR	Infrared
FT-IR	Fourier transform infrared spectroscopy
VCD	Vibrational circular dichroism
SEM	Scanning electron microscopy
XRPD	X-ray powder diffraction
DSC	Differential scanning calorimetry
BDS	Broadband dielectric spectroscopy
SPR	Surface plasmon resonance
UV–vis	Ultraviolet–visible
m.f.	Mole fraction
m.p.	Melting point
SD	Solid dispersion
PVP K30	Polyvinylpyrrolidone (Povidone K30)
BSA	Bovine serum albumin
DFT	Density functional theory
ISPA	Isolated spin-pair approximation
CD_3_CN	Trideuteroacetonitrile
C_6_D_6_	Deuterated benzene
DMSO-d_6_	Deuterated dimethyl sulfoxide
CDCl_3_	Deuterated chloroform
AN	Gutmann’s acceptor number
API	Active pharmaceutical ingredient
RESS	Rapid expansion of supercritical solutions
SAS	Supercritical anti-solvent
SBCW	Subcritical water
scCO_2_	Supercritical CO_2_
PEG	Polyethylene glycol
PLX	Poloxamer
NOE	Nuclear Overhauser effect
BZA	Benzamide
2OHBZA	Salicylamide

## References

[1] Bray F., Ferlay J., Soerjomataram I., Siegel R.L., Torre L.A., Jemal A. (2018). Global Cancer Statistics 2018: GLOBOCAN Estimates of Incidence and Mortality Worldwide for 36 Cancers in 185 Countries. CA Cancer J. Clin..

[2] Deng X., Shao Z., Zhao Y. (2021). Solutions to the Drawbacks of Photothermal and Photodynamic Cancer Therapy. Adv. Sci..

[3] Gavas S., Quazi S., Karpiński T.M. (2021). Nanoparticles for Cancer Therapy: Current Progress and Challenges. Nanoscale Res. Lett..

[4] Rawla P. (2019). Epidemiology of Prostate Cancer. World J. Oncol..

[5] Feldman B.J., Feldman D. (2001). The Development of Androgen-Independent Prostate Cancer. Nat. Rev. Cancer.

[6] Garje R., Chennamadhavuni A., Mott S.L., Chambers I.M., Gellhaus P., Zakharia Y., Brown J.A. (2020). Utilization and Outcomes of Surgical Castration in Comparison to Medical Castration in Metastatic Prostate Cancer. Clin. Genitourin. Cancer.

[7] Smith M.R., Hussain M., Saad F., Fizazi K., Sternberg C.N., Crawford E.D., Kopyltsov E., Park C.H., Alekseev B., Montesa-Pino Á. (2022). Darolutamide and Survival in Metastatic, Hormone-Sensitive Prostate Cancer. N. Engl. J. Med..

[8] Fizazi K., Foulon S., Carles J., Roubaud G., McDermott R., Fléchon A., Tombal B., Supiot S., Berthold D., Ronchin P. (2022). Abiraterone plus Prednisone Added to Androgen Deprivation Therapy and Docetaxel in de Novo Metastatic Castration-Sensitive Prostate Cancer (PEACE-1): A Multicentre, Open-Label, Randomised, Phase 3 Study with a 2 × 2 Factorial Design. Lancet.

[9] Armstrong A.J., Azad A.A., Iguchi T., Szmulewitz R.Z., Petrylak D.P., Holzbeierlein J., Villers A., Alcaraz A., Alekseev B., Shore N.D. (2022). Improved Survival with Enzalutamide in Patients with Metastatic Hormone-Sensitive Prostate Cancer. J. Clin. Oncol..

[10] James N.D., de Bono J.S., Spears M.R., Clarke N.W., Mason M.D., Dearnaley D.P., Ritchie A.W.S., Amos C.L., Gilson C., Jones R.J. (2017). Abiraterone for Prostate Cancer Not Previously Treated with Hormone Therapy. N. Engl. J. Med..

[11] Šlechta P., Viták R., Bárta P., Koucká K., Berková M., Žďárová D., Petríková A., Kuneš J., Kubíček V., Doležal M. (2024). Replacement of Nitro Function by Free Boronic Acid in Non-Steroidal Anti-Androgens. RSC Med. Chem..

[12] Vega D.R., Polla G., Martinez A., Mendioroz E., Reinoso M. (2007). Conformational Polymorphism in Bicalutamide. Int. J. Pharm..

[13] Korlyukov A.A., Malinska M., Vologzhanina A.V., Goizman M.S., Trzybinski D., Wozniak K. (2020). Charge Density View on Bicalutamide Molecular Interactions in the Monoclinic Polymorph and Androgen Receptor Binding Pocket. IUCrJ.

[14] Bruchovsky N., Wilson J.D. (1968). The Conversion of Testosterone to 5α-Androstan-17β-Ol-3-One by Rat Prostate in Vivo and in Vitro. Elsevier.

[15] Petrow V. (1986). The Dihydrotestosterone (DHT) Hypothesis of Prostate Cancer and Its Therapeutic Implications. Prostate.

[16] Wilson J.D. (1996). Role of Dihydrotestosterone in Androgen Action. Prostate.

[17] Huggins C. (1967). Endocrine-Induced Regression of Cancers. Science.

[18] Greenlee R.T., Murray T., Bolden S., Wingo P.A. (2000). Cancer Statistics, 2000. CA Cancer J. Clin..

[19] Payen O., Top S., Vessières A., Brulé E., Plamont M.A., McGlinchey M.J., Müller-Bunz H., Jaouen G. (2008). Synthesis and Structure-Activity Relationships of the First Ferrocenyl-Aryl-Hydantoin Derivatives of the Nonsteroidal Antiandrogen Nilutamide. J. Med. Chem..

[20] Slater S., Oliver R.T.D. (2000). Testosterone: Its Role in Development of Prostate Cancer and Potential Risk from Use as Hormone Replacement Therapy. Drugs Aging.

[21] Eaton N.E., Reeves G.K., Appleby P.N., Key T.J. (1999). Endogenous Sex Hormones and Prostate Cancer: A Quantitative Review of Prospective Studies. Br. J. Cancer.

[22] Andersson S.O., Adami H.O., Bergstrom R., Wide L. (1993). Serum Pituitary and Sex Steroid Hormone Levels in the Etiology of Prostatic Cancer—A Population-Based Case-Control Study. Br. J. Cancer.

[23] Culig Z., Hoffmann J., Erdel M., Eder I.E., Hobisch A., Hittmair A., Bartsch G., Utermann G., Schneider M.R., Parczyk K. (1999). Switch from Antagonist to Agonist of the Androgen Receptor Blocker Bicalutamide Is Associated with Prostate Tumour Progression in a New Model System. Br. J. Cancer.

[24] Gur M., Dil E., Akdeniz E., Cobanoglu U., Kalyoncu N.I., Topbas M., Ozyavuz R., Author C., Kalyoncu N.İ. (2024). The Toxic Effects on the Testis of Flutamide vs. Bicalutamide vs. Cyproterone Acetate: An Experimental Rat Study. New Trends Med. Sci..

[25] Chen C.S., Gao G.L., Ho D.R., Lin C.Y., Chou Y.T., Chen S.C., Huang M.C., Kao W.Y., Su J.G.J. (2021). Cyproterone Acetate Acts as a Disruptor of the Aryl Hydrocarbon Receptor. Sci. Rep..

[26] Russell N., Hoermann R., Cheung A.S., Zajac J.D., Grossmann M. (2022). Effects of Oestradiol Treatment on Hot Flushes in Men Undergoing Androgen Deprivation Therapy for Prostate Cancer: A Randomised Placebo-Controlled Trial. Eur. J. Endocrinol..

[27] Tan X., Namadchian M., Baghayeri M. (2023). Follow up of the Prostate Cancer Treatment Based on a Novel Sensing Method for Anti-Prostate Cancer Drug (Flutamide). Environ. Res..

[28] Pavlik T., Gudkova V., Razvolyaeva D., Pavlova M., Kostukova N., Miloykovich L., Kolik L., Konchekov E., Shimanovskii N. (2023). The Role of Autophagy and Apoptosis in the Combined Action of Plasma-Treated Saline, Doxorubicin, and Medroxyprogesterone Acetate on K562 Myeloid Leukaemia Cells. Int. J. Mol. Sci..

[29] La Vecchia M., Galanti D., Fazio I., Paratore R., Borsellino N. (2022). Megestrol Acetate for Heavily Pretreated Metastatic Castration-Resistant Prostate Cancer: An Old Answer for a New Problem. Case Rep. Oncol..

[30] Sanad M.H., Challan S.B., Essam H.M., Abdou F.Y., Farag A.B. (2025). Design of a Novel Complex 99mTc-Nilutamide as a Tracer for Prostate Cancer Disorder Detection in Mice. Radiochim. Acta.

[31] Keerthika Devi R., Ganesan M., Chen T.W., Chen S.M., Lou B.S., Ajmal Ali M., Al-Hemaid F.M., Li R.H. (2022). Gadolinium Vanadate Nanosheets Entrapped with 1D-Halloysite Nanotubes-Based Nanocomposite for the Determination of Prostate Anticancer Drug Nilutamide. J. Electroanal. Chem..

[32] Ueda T., Shiraishi T., Miyashita M., Kayukawa N., Gabata Y., Sako S., Ogura R., Fujihara A., Okihara K., Ukimura O. (2024). Apalutamide versus Bicalutamide in Combination with Androgen Deprivation Therapy for Metastatic Hormone Sensitive Prostate Cancer. Sci. Rep..

[33] Schellhammer P.F. (2002). An Evaluation of Bicalutamide in the Treatment of Prostate Cancer. Expert Opin. Pharmacother..

[34] Beebe-Dimmer J.L., Ruterbusch J.J., Bylsma L.C., Gillezeau C., Fryzek J., Schultz N.M., Flanders S.C., Barlev A., Heath E., Quek R.G.W. (2018). Patterns of Bicalutamide Use in Prostate Cancer Treatment: A U.S. Real-World Analysis Using the SEER-Medicare Database. Adv. Ther..

[35] Stanisławska I.J., Piwowarski J.P., Granica S., Kiss A.K. (2018). The Effects of Urolithins on the Response of Prostate Cancer Cells to Non-Steroidal Antiandrogen Bicalutamide. Phytomedicine.

[36] Charalabidis A., Sfouni M., Bergström C., Macheras P. (2019). The Biopharmaceutics Classification System (BCS) and the Biopharmaceutics Drug Disposition Classification System (BDDCS): Beyond Guidelines. Int. J. Pharm..

[37] Zhang X., Lionberger R.A., Davit B.M., Yu L.X. (2011). Utility of Physiologically Based Absorption Modeling in Implementing Quality by Design in Drug Development. AAPS J..

[38] Johnson T.N., Bonner J.J., Tucker G.T., Turner D.B., Jamei M. (2018). Development and Applications of a Physiologically-Based Model of Paediatric Oral Drug Absorption. Eur. J. Pharm. Sci..

[39] Kesisoglou F., Wu Y. (2008). Understanding the Effect of API Properties on Bioavailability through Absorption Modeling. AAPS J..

[40] Lloyd A., Penson D., Dewilde S., Kleinman L. (2007). Eliciting Patient Preferences for Hormonal Therapy Options in the Treatment of Metastatic Prostate Cancer. Prostate Cancer Prostatic Dis..

[41] Fradet Y. (2004). Bicalutamide (Casodex®) in the Treatment of Prostate Cancer. Expert Rev. Anticancer. Ther..

[42] Manso G., Thole Z., Salgueiro E., Revuelta P., Hidalgo A. (2006). Spontaneous Reporting of Hepatotoxicity Associated with Antiandrogens: Data from the Spanish Pharmacovigilance System. Pharmacoepidemiol. Drug Saf..

[43] Savjani K.T., Gajjar A.K., Savjani J.K. (2012). Drug Solubility: Importance and Enhancement Techniques. ISRN Pharm..

[44] Khadka P., Ro J., Kim H., Kim I., Kim J.T., Kim H., Cho J.M., Yun G., Lee J. (2014). Pharmaceutical Particle Technologies: An Approach to Improve Drug Solubility, Dissolution and Bioavailability. Asian J. Pharm. Sci..

[45] Khan A.D., Singh L. (2016). Various Techniques of Bioavailability Enhancement: A Review. J. Drug Deliv. Ther..

[46] Seedher N., Kanojia M. (2008). Micellar Solubilization of Some Poorly Soluble Antidiabetic Drugs: A Technical Note. AAPS PharmSciTech.

[47] Vandana K.R., Prasanna Raju Y., Harini Chowdary V., Sushma M., Vijay Kumar N. (2014). An Overview on in Situ Micronization Technique—An Emerging Novel Concept in Advanced Drug Delivery. Saudi Pharm. J..

[48] Han X., Ghoroi C., To D., Chen Y., Davé R. (2011). Simultaneous Micronization and Surface Modification for Improvement of Flow and Dissolution of Drug Particles. Int. J. Pharm..

[49] Loh Z.H., Samanta A.K., Sia Heng P.W. (2014). Overview of Milling Techniques for Improving the Solubility of Poorly Water-Soluble Drugs. Asian J. Pharm. Sci..

[50] Choudhury H., Gorain B., Madheswaran T., Pandey M., Kesharwani P., Tekade R.K. (2018). Drug Complexation: Implications in Drug Solubilization and Oral Bioavailability Enhancement. Dos. Form Des. Consid..

[51] Takenaka H., Kawashima Y., Lin S.Y., Ando Y. (1982). Preparations of Solid Particulates of Theophylline-ethylenediamine Complex by a Spray-drying Technique. J. Pharm. Sci..

[52] Brouwers J., Brewster M.E., Augustijns P. (2009). Supersaturating Drug Delivery Systems: The Answer to Solubility-Limited Oral Bioavailability?. J. Pharm. Sci..

[53] Hens B., Bermejo M., Tsume Y., Gonzalez-Alvarez I., Ruan H., Matsui K., Amidon G.E., Cavanagh K.L., Kuminek G., Benninghoff G. (2018). Evaluation and Optimized Selection of Supersaturating Drug Delivery Systems of Posaconazole (BCS Class 2b) in the Gastrointestinal Simulator (GIS): An in Vitro-in Silico-in Vivo Approach. Eur. J. Pharm. Sci..

[54] Vaksler Y.A., Benedis D., Dyshin A.A., Oparin R.D., Correia N.T., Capet F., Shishkina S.V., Kiselev M.G., Idrissi A. (2021). Spectroscopic Characterization of Single Co-Crystal of Mefenamic Acid and Nicotinamide Using Supercritical CO_2_. J. Mol. Liq..

[55] Singhal D., Curatolo W. (2004). Drug Polymorphism and Dosage Form Design: A Practical Perspective. Adv. Drug Deliv. Rev..

[56] Stanton M.K., Kelly R.C., Colletti A., Kiang Y.H., Langley M., Munson E.J., Peterson M.L., Roberts J., Wells M. (2010). Improved Pharmacokinetics of AMG 517 through Co-Crystallization Part 1: Comparison of Two Acids with Corresponding Amide Co-Crystals. J. Pharm. Sci..

[57] Nascimento A.L.C.S., Fernandes R.P., Charpentier M.D., ter Horst J.H., Caires F.J., Chorilli M. (2021). Co-Crystals of Non-Steroidal Anti-Inflammatory Drugs (NSAIDs): Insight toward Formation, Methods, and Drug Enhancement. Particuology.

[58] Kumar Bandaru R., Rout S.R., Kenguva G., Gorain B., Alhakamy N.A., Kesharwani P., Dandela R. (2021). Recent Advances in Pharmaceutical Cocrystals: From Bench to Market. Front. Pharmacol..

[59] Hu J., Johnston K.P., Williams R.O. (2004). Nanoparticle Engineering Processes for Enhancing the Dissolution Rates of Poorly Water Soluble Drugs. Drug Dev. Ind. Pharm..

[60] Chandel S., Bioavailability Enhancement A., Kumar Bolla P., Bhalani D.V., Nutan B., Kumar A., Singh Chandel A.K. (2022). Bioavailability Enhancement Techniques for Poorly Aqueous Soluble Drugs and Therapeutics. Biomedicines.

[61] Costa P., Sousa Lobo J.M. (2001). Modeling and Comparison of Dissolution Profiles. Eur. J. Pharm. Sci..

[62] Wei W., Evseenko V.I., Khvostov M.V., Borisov S.A., Tolstikova T.G., Polyakov N.E., Dushkin A.V., Xu W., Min L., Su W. (2021). Solubility, Permeability, Anti-Inflammatory Action and In Vivo Pharmacokinetic Properties of Several Mechanochemically Obtained Pharmaceutical Solid Dispersions of Nimesulide. Molecules.

[63] Aaltonen J., Allesø M., Mirza S., Koradia V., Gordon K.C., Rantanen J. (2008). Solid Form Screening—A Review. Eur. J. Pharm. Biopharm..

[64] Baghel S., Cathcart H., O’Reilly N.J. (2016). Polymeric Amorphous Solid Dispersions: A Review of Amorphization, Crystallization, Stabilization, Solid-State Characterization, and Aqueous Solubilization of Biopharmaceutical Classification System Class II Drugs. J. Pharm. Sci..

[65] Laitinen R., Löbmann K., Strachan C.J., Grohganz H., Rades T. (2013). Emerging Trends in the Stabilization of Amorphous Drugs. Int. J. Pharm..

[66] Liu L., Wang J.R., Mei X. (2022). Enhancing the Stability of Active Pharmaceutical Ingredients by the Cocrystal Strategy. CrystEngComm..

[67] Mannava M.K.C., Gunnam A., Lodagekar A., Shastri N.R., Nangia A.K., Solomon K.A. (2021). Enhanced Solubility, Permeability, and Tabletability of Nicorandil by Salt and Cocrystal Formation. CrystEngComm.

[68] Zhou Z., Li W., Sun W.J., Lu T., Tong H.H.Y., Sun C.C., Zheng Y. (2016). Resveratrol Cocrystals with Enhanced Solubility and Tabletability. Int. J. Pharm..

[69] Bolla G., Sarma B., Nangia A.K. (2022). Crystal Engineering of Pharmaceutical Cocrystals in the Discovery and Development of Improved Drugs. Chem. Rev..

[70] Wang J., Dai X.L., Lu T.B., Chen J.M. (2021). Temozolomide-Hesperetin Drug-Drug Cocrystal with Optimized Performance in Stability, Dissolution, and Tabletability. Cryst. Growth Des..

[71] Wong H.-H., Jessup A., Sertkaya A., Birkenbach A., Berlind A., Eyraud J. (2014). Examination of Clinical Trial Costs and Barriers for Drug Development Final.

[72] Sinha S., Vohora D. (2018). Drug Discovery and Development: An Overview. Pharm. Med. Transl. Clin. Res..

[73] Liberti L., Stolk P., McAuslane N., Somauroo A., Breckenridge A.M., Leufkens H.G.M. (2015). Adaptive Licensing and Facilitated Regulatory Pathways: A Survey of Stakeholder Perceptions. Clin. Pharmacol. Ther..

[74] Surov A.O., Ramazanova A.G., Voronin A.P., Drozd K.V., Churakov A.V., Perlovich G.L. (2023). Virtual Screening, Structural Analysis, and Formation Thermodynamics of Carbamazepine Cocrystals. Pharmaceutics.

[75] Karimi-Jafari M., Padrela L., Walker G.M., Croker D.M. (2018). Creating Cocrystals: A Review of Pharmaceutical Cocrystal Preparation Routes and Applications. Cryst. Growth Des..

[76] Joo H., Kraka E., Cremer D. (2008). Environmental Effects on Molecular Conformation: Bicalutamide Analogs. J. Mol. Struct. Theochem..

[77] Sobornova V.V., Belov K.V., Krestyaninov M.A., Khodov I.A. (2024). Influence of Solvent Polarity on the Conformer Ratio of Bicalutamide in Saturated Solutions: Insights from NOESY NMR Analysis and Quantum-Chemical Calculations. Int. J. Mol. Sci..

[78] Marhefka C.A., Gao W., Chung K., Kim J., He Y., Yin D., Bohl C., Dalton J.T., Miller D.D. (2004). Design, Synthesis, and Biological Characterization of Metabolically Stable Selective Androgen Receptor Modulators. J. Med. Chem..

[79] Hwang D.J., Yang J., Xu H., Rakov I.M., Mohler M.L., Dalton J.T., Miller D.D. (2006). Arylisothiocyanato Selective Androgen Receptor Modulators (SARMs) for Prostate Cancer. Bioorg. Med. Chem..

[80] Dhaked D.K., Jain V., Kasetti Y., Bharatam P.V. (2012). Conformational Polymorphism in Bicalutamide: A Quantum Chemical Study. Struct. Chem..

[81] Mukherjee A., Kirkovsky L., Yao X.T., Yates R.C., Miller D.D., Dalton J.T. (1996). Enantioselective Binding of Casodex to the Androgen Receptor. Xenobiotica.

[82] De Gaetano F., Cristiano M.C., Paolino D., Celesti C., Iannazzo D., Pistarà V., Iraci N., Ventura C.A. (2022). Bicalutamide Anticancer Activity Enhancement by Formulation of Soluble Inclusion Complexes with Cyclodextrins. Biomolecules.

[83] Mckillop D., Boyle G.W., Cockshott I.D., Jones D.C., Phillips P.J., Yates R.A. (1993). Metabolism and Enantioselective Pharmacokinetics of Casodex in Man. Xenobiotica.

[84] Kirkovsky L., Mukherjee A., Yin D., Dalton J.T., Miller D.D. (2000). Chiral Nonsteroidal Affinity Ligands for the Androgen Receptor. 1. Bicalutamide Analogues Bearing Electrophilic Groups in the B Aromatic Ring. J. Med. Chem..

[85] Nageswara Rao R., Narasa Raju A., Nagaraju D. (2006). An Improved and Validated LC Method for Resolution of Bicalutamide Enantiomers Using Amylose Tris-(3,5-Dimethylphenylcarbamate) as a Chiral Stationary Phase. J. Pharm. Biomed. Anal..

[86] Török R., Bor Á., Orosz G., Lukács F., Armstrong D.W., Péter A. (2005). High-Performance Liquid Chromatographic Enantioseparation of Bicalutamide and Its Related Compounds. J. Chromatogr. A.

[87] Sadutto D., Ferretti R., Zanitti L., Casulli A., Cirilli R. (2016). Analytical and Semipreparative High Performance Liquid Chromatography Enantioseparation of Bicalutamide and Its Chiral Impurities on an Immobilized Polysaccharide-Based Chiral Stationary Phase. J. Chromatogr. A.

[88] Osguthorpe D.J., Hagler A.T. (2011). Mechanism of Androgen Receptor Antagonism by Bicalutamide in the Treatment of Prostate Cancer. Biochemistry.

[89] Bohl C.E., Gao W., Miller D.D., Bell C.E., Dalton J.T. (2005). Structural Basis for Antagonism and Resistance of Bicalutamide in Prostate Cancer. Proc. Natl. Acad. Sci. USA.

[90] Blaising J., Polyak S.J., Pécheur E.I. (2014). Arbidol as a Broad-Spectrum Antiviral: An Update. Antiviral Res..

[91] Perlovich G.L., Blokhina S.V., Manin N.G., Volkova T.V., Tkachev V.V. (2013). Polymorphism and Solvatomorphism of Bicalutamide: Thermophysical Study and Solubility. J. Therm. Anal. Calorim..

[92] Bis J.A., Vishweshwar P., Weyna D., Zaworotko M.J. (2007). Hierarchy of Supramolecular Synthons: Persistent Hydroxyl···Pyridine Hydrogen Bonds in Cocrystals That Contain a Cyano Acceptor. Mol. Pharm..

[93] Surov A.O., Solanko K.A., Bond A.D., Bauer-Brandl A., Perlovich G.L. (2016). Cocrystals of the Antiandrogenic Drug Bicalutamide: Screening, Crystal Structures, Formation Thermodynamics and Lattice Energies. CrystEngComm.

[94] Hu X.R., Gu J.M. (2005). N-[4-Cyano-3-(Trifluoromethyl)Phenyl]-3-(4-Fluorophenylsulfonyl) -2-Hydroxy-2-Methylpropionamide. Acta Crystallogr. Sect. E Struct. Report Online.

[95] Nair V.A., Mustafa S.M., Mohler M.L., Dalton J.T., Miller D.D. (2006). Synthesis of Oxazolidinedione Derived Bicalutamide Analogs. Tetrahedron Lett..

[96] Mololina A.A., Sobornova V.V., Krestyaninov M.A., Belov K.V., Khodov I.A. (2024). Conformational Equilibria of Bicalutamide: A Study Based on One-Dimensional Selective Nuclear Overhauser Effect Experiments. Russ. J. Phys. Chem. A.

[97] Le Y., Ji H., Chen J.F., Shen Z., Yun J., Pu M. (2009). Nanosized Bicalutamide and Its Molecular Structure in Solvents. Int. J. Pharm..

[98] Miertuš S., Scrocco E., Tomasi J. (1981). Electrostatic Interaction of a Solute with a Continuum. A Direct Utilizaion of AB Initio Molecular Potentials for the Prevision of Solvent Effects. Chem. Phys..

[99] Pascual-ahuir J.L., Silla E., Tuñon I., Pascual-ahuir J.L., Silla E., Tuñon I. (1994). GEPOL: An Improved Description of Molecular Surfaces. III. A New Algorithm for the Computation of a Solvent-Excluding Surface. J. Comput. Chem..

[100] Volkova T.V., Simonova O.R., Perlovich G.L. (2022). Physicochemical Profile of Antiandrogen Drug Bicalutamide: Solubility, Distribution, Permeability. Pharmaceutics.

[101] Escudero F., Yáñez M. (1982). Atoms in Molecules. Mol. Phys..

[102] Chakalov E.R., Tupikina E.Y., Ivanov D.M., Bartashevich E.V., Tolstoy P.M. (2022). The Distance between Minima of Electron Density and Electrostatic Potential as a Measure of Halogen Bond Strength. Molecules.

[103] Mololina A.A., Sobornova V.V., Belov K.V., Krestyaninov M.A., Khodov I.A. (2025). Role of Non-Covalent Interactions in the Conformational Stability of Bicalutamide in Different Solvent Environments: Insights from Quantum-Chemical Calculations and NMR Spectroscopy. J. Mol. Liq..

[104] Belov K.V., Khodov I.A. (2025). NOESY Investigation of Bicalutamide Conformational Changes in DMSO: Role of Solution Concentration in Polymorph Formation. J. Mol. Liq..

[105] Rams-Baron M., Wlodarczyk P., Dulski M., Wlodarczyk A., Kruk D., Rachocki A., Jachowicz R., Paluch M. (2017). The Indications of Tautomeric Conversion in Amorphous Bicalutamide Drug. Eur. J. Pharm. Sci..

[106] Ren F., Jing Q., Tang Y., Shen Y., Chen J., Gao F., Cui J. (2006). Characteristics of Bicalutamide Solid Dispersions and Improvement of the Dissolution. Drug Dev. Ind. Pharm..

[107] Cheng X., Chen X., Liang C., Jin H., Ren S., Xue R., Chen F. (2023). Explanation and Prediction for the Crystallized Products from Amorphous Bicalutamide and Bicalutamide Solutions by Using Mid-Frequency Raman Difference Spectra. Vib. Spectrosc..

[108] Tres F., Patient J.D., Williams P.M., Treacher K., Booth J., Hughes L.P., Wren S.A.C., Aylott J.W., Burley J.C. (2015). Monitoring the Dissolution Mechanisms of Amorphous Bicalutamide Solid Dispersions via Real-Time Raman Mapping. Mol. Pharm..

[109] Ray S., Ghosh S., Mandal S. (2017). Development of Bicalutamide-Loaded PLGA Nanoparticles: Preparation, Characterization and in-Vitro Evaluation for the Treatment of Prostate Cancer. Artif. Cells Nanomedicine Biotechnol..

[110] Yang C., Di P., Fu J.P., Xiong H., Jing Q., Ren G., Tang Y., Zheng W., Liu G., Ren F. (2017). Improving the Physicochemical Properties of Bicalutamide by Complex Formation with Bovine Serum Albumin. Eur. J. Pharm. Sci..

[111] Szafraniec-Szczęsny J., Antosik-Rogóż A., Knapik-Kowalczuk J., Kurek M., Szefer E., Gawlak K., Chmiel K., Peralta S., Niwiński K., Pielichowski K. (2020). Compression-Induced Phase Transitions of Bicalutamide. Pharmaceutics.

[112] Szczurek J., Rams-Baron M., Knapik-Kowalczuk J., Antosik A., Szafraniec J., Jamróz W., Dulski M., Jachowicz R., Paluch M. (2017). Molecular Dynamics, Recrystallization Behavior, and Water Solubility of the Amorphous Anticancer Agent Bicalutamide and Its Polyvinylpyrrolidone Mixtures. Mol. Pharm..

[113] Cruz-Cabeza A.J., Bernstein J. (2014). Conformational Polymorphism. Chem. Rev..

[114] Nangia A. (2008). Conformational Polymorphism in Organic Crystals. Acc. Chem. Res..

[115] Bernstein J., Hagler A.T. (1978). Conformational Polymorphism. The Influence of Crystal Structure on Molecular Conformation. J. Am. Chem. Soc..

[116] Bauer J., Spanton S., Henry R., Quick J., Dziki W., Porter W., Morris J. (2001). Ritonavir: An Extraordinary Example of Conformational Polymorphism. Pharm. Res..

[117] Shi P., Han Y., Zhu Z., Gong J. (2023). Research Progress on the Molecular Mechanism of Polymorph Nucleation in Solution: A Perspective from Research Mentality and Technique. Crystals.

[118] Yu L., Reutzel-Edens S.M., Mitchell C.A. (2000). Crystallization and Polymorphism of Conformationally Flexible Molecules: Problems, Patterns, and Strategies. Org. Process Res. Dev..

[119] Shukla A.K., Maikap G.C., Agarwal S.K. (2006). Process for Preparation of Bicalutamide.

[120] Shintaku T., Katsura T., Itaya N. (2004). Crystal of Bicalutamide and Production Method Thereof.

[121] Perlovich G.L., Bauer-Brandl A. (2008). Solvation of Drugs as a Key for Understanding Partitioning and Passive Transport Exemplified by NSAIDs. Curr. Drug Deliv..

[122] Német Z., Sztatisz J., Demeter Á. (2008). Polymorph Transitions of Bicalutamide: A Remarkable Example of Mechanical Activation. J. Pharm. Sci..

[123] Ito T., Byrn S., Chen X., Carvajal M.T. (2011). Thermal Insight of Mechanically Activated Bile Acid Powders. Int. J. Pharm..

[124] Li X., Wang N., Yang J., Huang Y., Ji X., Huang X., Wang T., Wang H., Hao H. (2020). Molecular Conformational Evolution Mechanism during Nucleation of Crystals in Solution. IUCrJ.

[125] Back K.R., Davey R.J., Grecu T., Hunter C.A., Taylor L.S. (2012). Molecular Conformation and Crystallization: The Case of Ethenzamide. Cryst. Growth Des..

[126] Shi P., Xu S., Du S., Rohani S., Liu S., Tang W., Jia L., Wang J., Gong J. (2018). Insight into Solvent-Dependent Conformational Polymorph Selectivity: The Case of Undecanedioic Acid. Cryst. Growth Des..

[127] Oparin R.D., Vaksler Y.A., Krestyaninov M.A., Idrissi A., Shishkina S.V., Kiselev M.G. (2019). Polymorphism and Conformations of Mefenamic Acid in Supercritical Carbon Dioxide. J. Supercrit. Fluids.

[128] Belov K.V., Dyshin A.A., Krestyaninov M.A., Efimov S.V., Khodov I.A., Kiselev M.G. (2022). Conformational Preferences of Tolfenamic Acid in DMSO-CO_2_ Solvent System by 2D NOESY. J. Mol. Liq..

[129] Khodov I.A., Belov K.V., Krestyaninov M.A., Dyshin A.A., Kiselev M.G., Krestov G.A. (2023). Investigation of the Spatial Structure of Flufenamic Acid in Supercritical Carbon Dioxide Media via 2D NOESY. Materials.

[130] Yamasaki R., Tanatani A., Azumaya I., Masu H., Yamaguchi K., Kagechika H. (2006). Solvent-Dependent Conformational Switching of N-Phenylhydroxamic Acid and Its Application in Crystal Engineering. Cryst. Growth Des..

[131] Ischenko V., Englert U., Jansen M. (2005). Conformational Dimorphism of 1,1,3,3,5,5-Hexachloro-1,3,5-Trigermacyclohexane: Solvent-Induced Crystallization of a Metastable Polymorph Containing Boat-Shaped Molecules. Chem. A Eur. J..

[132] Gutmann V., Wychera E. (1966). Coordination Reactions in Non Aqueous Solutions—The Role of the Donor Strength. Inorg. Nucl. Chem. Lett..

[133] Mayer U., Gutmann V., Gerger W. (1975). The Acceptor Number—A Quantitative Empirical Parameter for the Electrophilic Properties of Solvents. Monatshefte Chem..

[134] Knez Ž., Markočič E., Leitgeb M., Primožič M., Hrnčič M.K., Škerget M. (2014). Industrial Applications of Supercritical Fluids: A Review. Energy.

[135] Girotra P., Singh S.K., Nagpal K. (2013). Supercritical Fluid Technology: A Promising Approach in Pharmaceutical Research. Pharm. Dev. Technol..

[136] Li K., Xu Z. (2019). A Review of Current Progress of Supercritical Fluid Technologies for E-Waste Treatment. J. Clean. Prod..

[137] Preetam A., Jadhao P.R., Naik S.N., Pant K.K., Kumar V. (2023). Supercritical Fluid Technology—An Eco-Friendly Approach for Resource Recovery from e-Waste and Plastic Waste: A Review. Sep. Purif. Technol..

[138] de Oliveira C.R.S., de Oliveira P.V., Pellenz L., de Aguiar C.R.L., da Silva Júnior A.H. (2024). Supercritical Fluid Technology as a Sustainable Alternative Method for Textile Dyeing: An Approach on Waste, Energy, and CO_2_ Emission Reduction. J. Environ. Sci..

[139] Fraguela-Meissimilly H., Bastías-Monte J.M., Vergara C., Ortiz-Viedma J., Lemus-Mondaca R., Flores M., Toledo-Merma P., Alcázar-Alay S., Gallón-Bedoya M. (2023). New Trends in Supercritical Fluid Technology and Pressurized Liquids for the Extraction and Recovery of Bioactive Compounds from Agro-Industrial and Marine Food Waste. Molecules.

[140] Liu Z., Navik R., Tan H., Xiang Q., Wahyudiono, Goto M., Ibarra R.M., Zhao Y. (2022). Graphene-Based Materials Prepared by Supercritical Fluid Technology and Its Application in Energy Storage. J. Supercrit. Fluids.

[141] Zhou J., Gullón B., Wang M., Gullón P., Lorenzo J.M., Barba F.J. (2021). The Application of Supercritical Fluids Technology to Recover Healthy Valuable Compounds from Marine and Agricultural Food Processing By-Products: A Review. Processes.

[142] Tran P., Park J.S. (2021). Application of Supercritical Fluid Technology for Solid Dispersion to Enhance Solubility and Bioavailability of Poorly Water-Soluble Drugs. Int. J. Pharm..

[143] Ranieri U., Formisano F., Gorelli F.A., Santoro M., Koza M.M., De Francesco A., Bove L.E. (2024). Crossover from Gas-like to Liquid-like Molecular Diffusion in a Simple Supercritical Fluid. Nat. Commun..

[144] Du Y., Liao G., Zhang F., Jiaqiang E., Chen J. (2024). Recognition of Supercritical CO_2_ Liquid-like and Gas-like Molecules Based on Deep Neural Network. J. Supercrit. Fluids.

[145] Carlès P. (2010). A Brief Review of the Thermophysical Properties of Supercritical Fluids. J. Supercrit. Fluids.

[146] King J.W. (2014). Modern Supercritical Fluid Technology for Food Applications. Annu. Rev. Food Sci. Technol..

[147] Braga M.E.M., Gaspar M.C., de Sousa H.C. (2023). Supercritical Fluid Technology for Agrifood Materials Processing. Curr. Opin. Food Sci..

[148] Abdulagatov I.M., Skripov P.V. (2021). Thermodynamic and Transport Properties of Supercritical Fluids. Part 2: Review of Transport Properties. Russ. J. Phys. Chem. B.

[149] Martín A., Cocero M.J. (2008). Micronization Processes with Supercritical Fluids: Fundamentals and Mechanisms. Adv. Drug Deliv. Rev..

[150] Kerč J., Srčič S., Knez Ž., Senčar-Božič P. (1999). Micronization of Drugs Using Supercritical Carbon Dioxide. Int. J. Pharm..

[151] Türk M. (2022). Particle Synthesis by Rapid Expansion of Supercritical Solutions (RESS): Current State, Further Perspectives and Needs. J. Aerosol Sci..

[152] Liu G., Li J., Deng S. (2021). Applications of Supercritical Anti-Solvent Process in Preparation of Solid Multicomponent Systems. Pharmaceutics.

[153] Kayrak D., Akman U., Hortaçsu Ö. (2003). Micronization of Ibuprofen by RESS. J. Supercrit. Fluids.

[154] Pathak P., Meziani M.J., Desai T., Sun Y.P. (2006). Formation and Stabilization of Ibuprofen Nanoparticles in Supercritical Fluid Processing. J. Supercrit. Fluids.

[155] Jung J., Perrut M. (2001). Particle Design Using Supercritical Fluids: Literature and Patent Survey. J. Supercrit. Fluids.

[156] Knez Ž., Hrnčič M.K., Škerget M. (2015). Particle Formation and Product Formulation Using Supercritical Fluids. Annu. Rev. Chem. Biomol. Eng..

[157] Jessop P.G., Subramaniam B. (2007). Gas-Expanded Liquids. Chem. Rev..

[158] Rossmann M., Braeuer A., Dowy S., Gallinger T.G., Leipertz A., Schluecker E. (2012). Solute Solubility as Criterion for the Appearance of Amorphous Particle Precipitation or Crystallization in the Supercritical Antisolvent (SAS) Process. J. Supercrit. Fluids.

[159] Chong G.H., Yunus R., Choong T.S.Y., Abdullah N., Spotar S.Y. (2011). Simple Guidelines for a Self-Built Laboratory-Scale Supercritical Anti-Solvent System. J. Supercrit. Fluids.

[160] Pu Y., Cai F., Wang D., Li Y., Chen X., Maimouna A.G., Wu Z., Wen X., Chen J.F., Foster N.R. (2017). Solubility of Bicalutamide, Megestrol Acetate, Prednisolone, Beclomethasone Dipropionate, and Clarithromycin in Subcritical Water at Different Temperatures from 383.15 to 443.15 K. J. Chem. Eng. Data.

[161] Szafraniec J., Antosik A., Knapik-Kowalczuk J., Kurek M., Syrek K., Chmiel K., Paluch M., Jachowicz R. (2017). Planetary Ball Milling and Supercritical Fluid Technology as a Way to Enhance Dissolution of Bicalutamide. Int. J. Pharm..

[162] Patel S., Kou X., Hou H., Huang Y., Strong J.C., Zhang G.G.Z., Sun C.C. (2017). Mechanical Properties and Tableting Behavior of Amorphous Solid Dispersions. J. Pharm. Sci..

[163] Antosik-Rogóż A., Szafraniec-Szczęsny J., Gawlak K., Knapik-Kowalczuk J., Paluch M., Jachowicz R. (2020). Tabletting Solid Dispersions of Bicalutamide Prepared Using Ball-Milling or Supercritical Carbon Dioxide: The Interrelationship between Phase Transition and in-Vitro Dissolution. Pharm. Dev. Technol..

[164] Antosik-Rogóż A., Szafraniec-Szczęsny J., Chmiel K., Knapik-Kowalczuk J., Kurek M., Gawlak K., Danesi V.P., Paluch M., Jachowicz R. (2020). How Does the CO_2_ in Supercritical State Affect the Properties of Drug-Polymer Systems, Dissolution Performance and Characteristics of Tablets Containing Bicalutamide?. Materials.

[165] Belov K.V., Khodov I.A. (2025). Open or Closed? Quantifying Bicalutamide Conformers in Supercritical Carbon Dioxide by 2D NOESY. J. Mol. Struct..

[166] Belov K.V., Dyshin A.A., Krestyaninov M.A., Khodov I.A. (2025). 1D NOESY Study of Bicalutamide Conformations in a Supercritical CO_2_. J. Mol. Liq..

[167] Pasquali I., Bettini R., Giordano F. (2008). Supercritical Fluid Technologies: An Innovative Approach for Manipulating the Solid-State of Pharmaceuticals. Adv. Drug Deliv. Rev..

[168] Moribe K., Tozuka Y., Yamamoto K. (2008). Supercritical Carbon Dioxide Processing of Active Pharmaceutical Ingredients for Polymorphic Control and for Complex Formation. Adv. Drug Deliv. Rev..

[169] Tan H.S., Borsadia S. (2001). Particle Formation Using Supercritical Fluids: Pharmaceutical Applications. Expert Opin. Ther. Pat..

